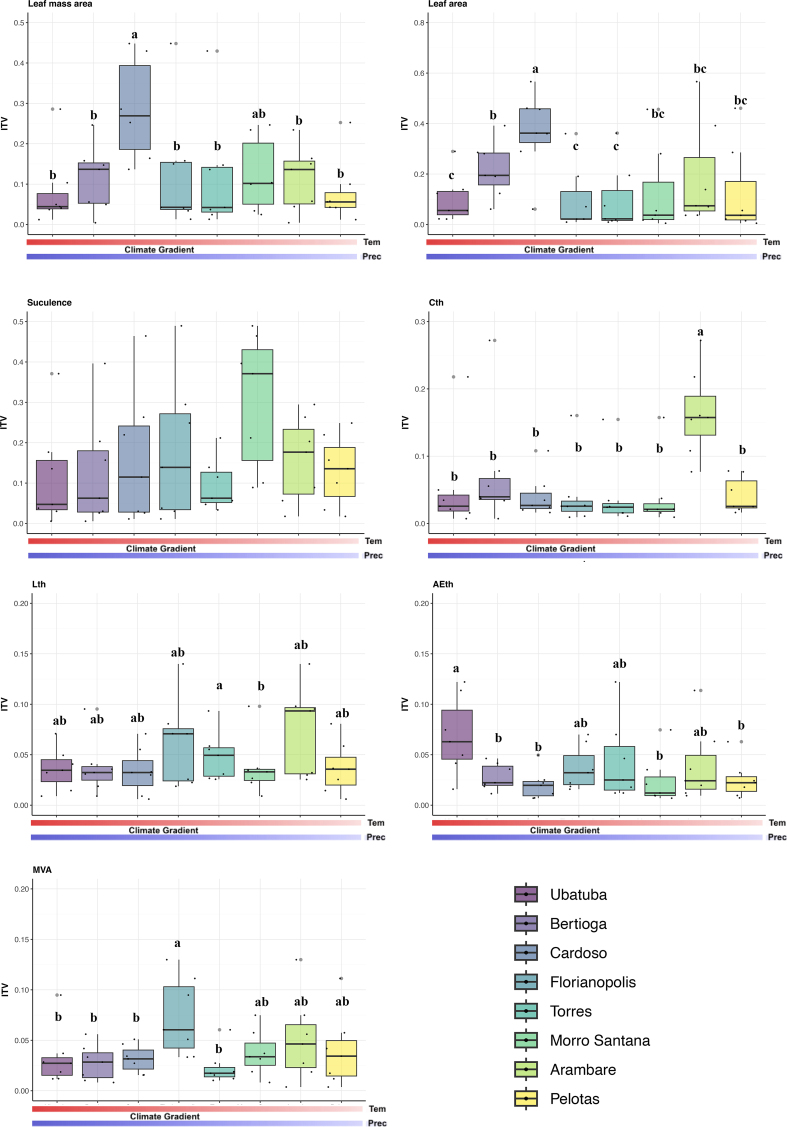# We are not all the same: The role of intrapopulation trait variability in shaping functional strategy and performance of widespread species

**DOI:** 10.1016/j.pld.2025.06.008

**Published:** 2025-06-30

**Authors:** Welington L. Sachetti, Vitor de A. Kamimura, Juliana L.S. Mayer, Beatriz L. Arida, Thales M. de Lima, Diego S. Graciano, Fábio Pinheiro

**Affiliations:** a Departamento de Biologia Vegetal, Universidade Estadual de Campinas (UNICAMP), Rua Monteiro Lobato, 255, Cidade Universitária, Campinas, São Paulo, 13083-862, Brazil; b Royal Botanic Garden Edinburgh, Edinburgh EH3 5LR, United Kingdom; c Institute of Ecology and Evolution, School of Biological Sciences, University of Edinburgh, Edinburgh EH9 3FL, United Kingdom

**Keywords:** Latitudinal gradient, Phenotype, Plant adaptability, Plant population, Reproduction, Species interactions

## Abstract

Functional traits influence plant performance and adaptability to environmental changes, as environments select optimal phenotypes, leading to diverse functional strategies. While trait-based studies emphasize intraspecific trait variability (ITV) in response to environmental variations, the effects of phenotype–environment relationships and ITV on the performance of widely distributed species remain unclear. Here, we evaluated how intraspecific phenotypic dissimilarity (IPD) between populations, ITV within populations, and biotic and abiotic factors influence the functional strategies and performance of eight *Epidendrum fulgens* populations along a latitudinal gradient on the Brazilian coast. Data on seven functional traits (leaf and anatomical) and performance (fruit set over two years) were collected, and biotic and abiotic factors were analyzed using bioclimatic variables and phylogenetic structures of plant communities. The functional space was evaluated using PCA, and GLMs were employed to assess the impacts of environmental factors, species interactions, IPD, and ITV on performance. Populations exhibited distinct functional strategies, with warmer, wetter environments favoring acquisitive strategies and colder, drier areas favoring conservative ones. Notably, IPD and, unexpectedly, were correlated with improved performance, with IPD mitigating temperature stress. ITV within populations had significant but context-dependent effects on outcomes. In summary, our findings highlight the role of intrapopulation trait variability in driving shifts in functional strategies across environmental gradients, improving performance through increased phenotypic dissimilarity. The interplay of ITV within populations, environmental conditions, and interspecific competition shapes plant strategies and performance across diverse habitats.

## Highlights

• Testing the influence of phenotype–environment relationships and intraspecific trait variability (ITV) on the performance of widely distributed species.
•Different levels of environmental stress drive the selection of either more conservative or more acquisitive strategies.
•Greater phenotypic dissimilarity improves *Epidendrum fulgens* performance even under harsh conditions.
•The impact of intrapopulation trait variability on species performance is trait-specific and context-dependent.
•Intrapopulation trait variability unveils the interplay among multidimensional phenotypes, species interactions, and performance.

## 1 Introduction

Functional traits are crucial in determining the performance of plant species (Violle et al., 2007, 2012) and their ability to adapt to changing environmental conditions (Sobral, 2021). Species with broad geographic distributions have often served as models for understanding how environmental gradients shape species trait variability (Clausen et al., 1948; Westerband et al., 2021). Trait-based approaches have highlighted the responsiveness of traits to environmental variations, focusing on differences between populations and species levels (Kumordzi et al., 2014; Carmona et al., 2019; Kitagawa et al., 2022). However, significant trait variability also exists within populations (Albert et al., 2010, 2011; Helsen et al., 2017; Westerband et al., 2021). Recent advances in understanding the drivers of intraspecific trait variability (ITV) support both the stress-reduced and stress-induced variability hypotheses (Valladares et al., 2007; Siefert et al., 2015; Helsen et al., 2017). In particular, Puglielli et al. (2024) showed the contribution of ITV to leaf functional strategies is strongly dependent on habitat and trait identity, with different environmental filters selecting for contrasting ITV patterns. Although these findings highlight the complex nature of trait adaptation (Helsen et al., 2017; Kuppler et al., 2020), the variation in intraspecific phenotype–environment relationships and the role of within-population ITV in shaping the performance of widely distributed species remain poorly understood.

The environment, through abiotic and biotic pressures, selects the optimal phenotype suited to a specific range of resources and conditions, such as climate and nutrient availability in different populations (Violle et al., 2007; Helsen et al., 2017). Consequently, different abiotic and biotic conditions across environmental gradients tend to select diverse ecological strategies among individuals of the same species (Helsen et al., 2017). The phenotype of an individual is an expression of multiple integrated traits that are jointly influenced by genetic, environmental, and developmental effects (Pigliucci, 2003). Functional traits often involve trade-offs influenced by local environmental conditions, which also affect how traits are combined (Bruelheide et al., 2018; Kamimura et al., 2023). These interactions drive shifts in trait–performance relationships along environmental gradients, and functional trait space analyses are effective tools for capturing this intraspecific phenotypic variability (Carmona et al., 2024). These analyses reveal how different habitats modulate the effects of individual traits and their interactions on species performance under stress or competition (Laughlin and Messier, 2015; Puglielli et al., 2024; Yang et al., 2024). Functional spaces are typically used to differentiate species or groups (e.g., Díaz et al., 2016; Carmona et al., 2021), but they also offer valuable insights into population-level variation within widespread species. Distinct phenotypes can represent alternative functional strategies with similar performance outcomes (Laughlin and Messier, 2015; Dias et al., 2020), emphasizing the importance of considering alternative functional design (i.e. combinations of traits representing adaptive strategies) to better understand phenotype–environment–performance relationships (Dias et al., 2020; Kamimura et al., 2023).

Both intra- and interspecific variability in functional traits is shaped by abiotic and biotic factors, such as species competition, often exceeding previous expectations (di Biase et al., 2021). Spatial variability in climatic conditions further drives differentiation within plant populations (Carlucci et al., 2015). ITV is a key indicator of a species’ adaptive potential to environmental gradients and biotic interactions, particularly in widely distributed populations (Violle et al., 2012; Carlucci et al., 2015; Westerband et al., 2021). For example, ITV in leaf traits, such as leaf area and leaf mass per area, directly influences vital processes like photosynthesis and water use efficiency (Ackerly et al., 2000; Wright et al., 2004; Poorter et al., 2009; Pérez-Harguindeguy et al., 2016), thereby affecting growth and reproduction (Lambers et al., 2008; Cornelissen, 1999; Kuppler et al., 2020). Although several studies have examined ITV (Westerband et al., 2021), the interplay among ITV, species competition, and environmental gradients remains underexplored. Assessing the phylogenetic relatedness of cooccurring species can reveal how evolutionary history influences ecological strategies under varying climatic and biotic conditions (Webb et al., 2002; Mayfield and Levine, 2010; Massante et al., 2019; Bergamin et al., 2021; Fan et al., 2023), in that way addressing the ongoing challenge of elucidating how ITV, together with biotic and abiotic filters, shapes species performance and distribution.

Here we investigated the role of intraspecific phenotypic dissimilarity among and within populations (hereafter IPD, a measure for intraspecific phenotypic variability), ITV within populations, and abiotic and biotic interactions on the functional strategies and performance of *Epidendrum fulgens* (Orchidaceae), a species widely distributed along the south and southeast coasts of Brazil (Pinheiro et al., 2011; de Mattos et al., 2023). Specifically, we explored: (i) whether distinct populations along a latitudinal gradient show different functional strategies, and (ii) how IPD, ITV within populations, interspecific competition, and climatic factors affect population performance. Our comprehensive data set includes a fruit set and seven leaf functional traits of individuals from eight different populations of *E*. *fulgens*. We hypothesize that trade-offs in functional traits lead to different resource acquisition and allocation strategies among populations, with harsh environments promoting conservative strategies (Violle et al., 2007; Díaz et al., 2016). We expect to find both stress-reduced and stress-induced variability in populations along the species latitudinal gradient (Helsen et al., 2017; Kuppler et al., 2020), with greater IPD and higher ITV within populations related to better performance, mitigating effects of interspecific competition and harsh environmental conditions (Dias et al., 2020; Kamimura et al., 2023; Yang et al., 2024).

## 2 Methods

### 2.1 Study species and site

We studied eight populations of *Epidendrum fulgens* (Table S1), an endemic orchid species from Brazil, specifically from the sandy coastal regions known as *Restingas* and rock outcrops. *E*. *fulgens* has a wide distribution, occurring along the southeast and southern Brazilian coast across different biomes. *E*. *fulgens* is found within the Atlantic Forest and Pampas biomes (Fig. 1), spanning approximately 1200 km (de Mattos et al., 2023). Along this latitudinal gradient, mean annual temperatures range from 22.5 °C in the north to 12 °C in the south, and mean annual precipitation decreases from 3000 mm to 1500 mm. *Restingas* are low-lying coastal plains, typically less than 10 m above sea level, characterized by sandy soils and herbaceous and shrub vegetation. These areas face harsh environmental conditions, such as droughts, extreme temperatures, salinity, nutrient scarcity, and constant winds (Scarano, 2002). Granite rock outcrop formations experience environmental conditions similar to those in *Restingas*, except for elevated salinity.

**Fig. 1:**
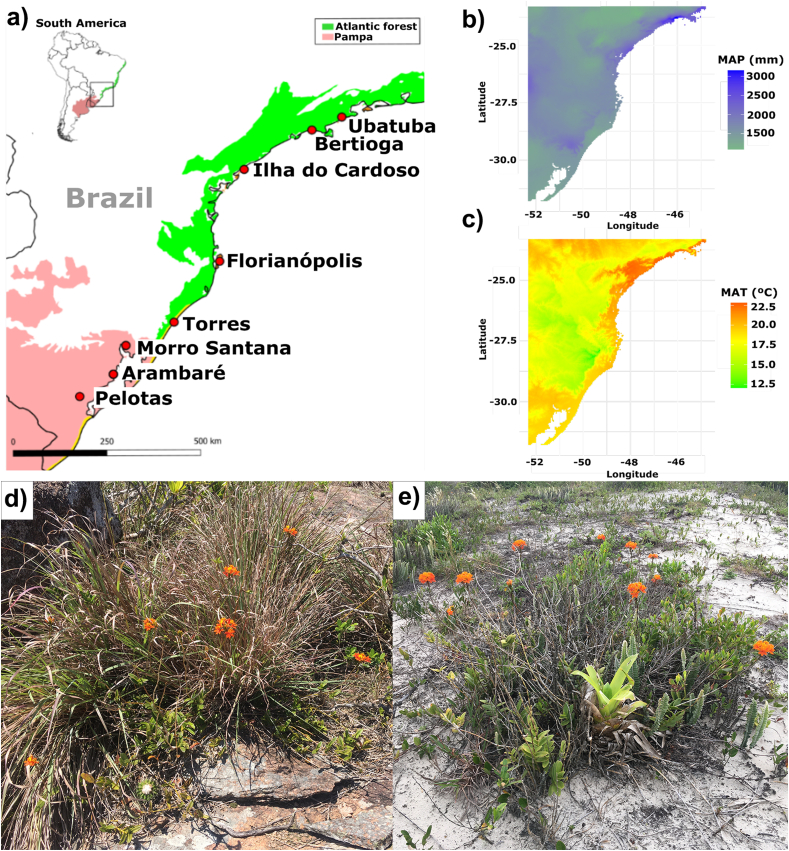
Map showing the locations of the *Epidendrum fulgens* populations studied (red dots) along the Brazilian coast, along with the environmental characteristics of the sampling sites. Mean annual precipitation (MAP - b) is color-coded from green (lower precipitation) to blue (higher precipitation), while mean annual temperature (MAT - c) is color-coded from green (12–15 °C) to orange (20–22 °C) along the latitudinal gradient of *Epidendrum fulgens* distribution in the study area. The populations at Morro Santana and Pelotas are found in rocky outcrops (d), whereas the other populations are located in sandy dune ecosystems (*Restingas* - e).

### 2.2 Data sampling on functional traits and performance of populations

We assessed functional traits using a standardized sampling protocol across all populations. In each population, we selected 10 individuals and collected a total of three fully expanded, healthy leaves per individual, free from signs of herbivory or disease. These leaves were used exclusively for measuring seven functional traits—three morphological and four anatomical—that are expected to respond to environmental conditions and relate to individual performance (Table 1).

**Table 1:** Methods for obtaining functional leaf traits and their ecological prediction.

Functional Trait	Methods	Ecological Prediction
Leaf area (LA)	The leaves were scanned using a known scale for calibration, and the one-sided area of the adaxial surface was measured.	The interspecific variation in LA has been associated with thermal stress, cold stress, drought stress, nutritional stress, high-radiation stress and seed mass, all of which favor relatively small leaves. (Cornelissen, 1999; Pérez-Harguindeguy et al., 2016).
Leaf mass per area (LMA)	The leaves were scanned, and their one-sided area was measured, followed by drying in an oven at 60 °C for two days or until the mass stabilized (LMA = dry mass/area).	A high LMA is associated with longer-lived leaves, a proxy for resource acquisition, which may be a strategy for survival in more stressful environments. Species with low LMA also tend to have higher photosynthetic capacity per unit leaf mass (Westoby et al., 2002)
Leaf succulence (LS)	Four leaf disks with a 1 cm diameter were obtained from the leaf blade, excluding the midrib and leaf edge, soaked in distilled water for 24 h, and their turgid mass was measured. Subsequently, the disks were dried in an oven at 60 °C for two days or until the mass stabilized (succulence = turgid mass–dry mass/area).	High succulence has been associated with regions of low, seasonal rainfall. Succulence can also be correlated with the presence of salt, with the purpose of the plant dealing with physiological drought (Males, 2017)
Leaf thickness (Lth)	The leaf thickness was measured at 1 mm from the main vascular bundle, incorporating the mesophyll thickness and the height of the abaxial and adaxial epidermis from anatomical sections.	The Lth was negatively correlated with air temperature and light, and positively correlated with ambient relative humidity (Syvertsen and Levy, 1982; Búrquez, 1987; Rozema et al., 1987).
Adaxial epidermis thickness (AEth)	The thickness of the adaxial epidermis was obtained from the average measurement of 10 equidistant cells from anatomical sections.	The AEth plays a significant role in leaf stiffness and is associated with perennial plants (Onoda et al., 2012), in addition to contributing to water storage (Zhang et al., 2018).
Metaxylem vessel area (MVA)	To obtain the area of the metaxylem vessels from anatomical sections, we measured the area of the three largest vessels in the main vein of the leaf, and then calculated the average.	The MVA is related to water transport, where vessels with smaller areas tend to provide greater hydraulic safety against embolism for the plant (Blackman et al., 2018).
Adaxial cuticle thickness (Cth)	For cuticle thickness, which was stained with Sudan IV for better visualization, 10 equidistant measurements were taken along 500 μm after the main vascular bundle, and the average was obtained.	A thicker cuticle provides greater tear resistance, as well as protection against solar radiation and water loss (Kosma et al., 2009; Onoda et al., 2012).

#### 2.2.1 Morphological leaf trait

Two leaves per individual were used to measure morphological traits. One leaf was used to assess leaf succulence (LS), while the other was used to determine leaf area (LA) and leaf mass per area (LMA). All morphological traits were measured following standardized protocols outlined by Pérez-Harguindeguy et al. (2016).

#### 2.2.2 Anatomical leaf traits

Anatomical traits were measured on the third leaf collected from each of the same individuals, ensuring consistency across all trait measurements. We measured four anatomical traits (Fig. S1): leaf thickness (LTh), adaxial cuticle thickness (CTh), adaxial epidermis thickness (AETh), and metaxylem vessel area (MVA), all of which are also expected to reflect environmental influences and relate to individual performance (Table 1). Leaves were fixed in the FAA solution (formaldehyde, acetic acid, 50% ethanol; 1:1:18 v/v) for 24 h, then stored in 70% ethanol. Samples were dehydrated in an ethanol gradient and embedded in hydroxyethyl-methacrylate (Historesin®, Leica), following the manufacturer's instructions. Cross-sections (5 μm thick) were obtained using a rotary microtome (Microm HM340E, Thermo Fisher Scientific Inc., Waltham, MA, USA). Sections were stained with 0.05% Toluidine Blue in citrate buffer (pH 4.5) (O'Brien et al., 1964) and Sudan IV for cuticle visualization (Jensen, 1962), then mounted in Entellan® synthetic resin (Merck KGaA, Darmstadt, Germany). Images were captured using a digital camera (Olympus DP71) attached to an optical microscope (Olympus BX51, Olympus Optical Co., Ltd., Japan).

#### 2.2.3 Performance of populations

To evaluate the performance of the *Epidendrum fulgens* population, we collected fruit count data from 15 to 20 individuals per population. Field data were gathered between November 2020 and January 2022, during five visits in the peak flowering period (November to March). Reproductive performance, measured as a fruit set, was used as an indicator of reproductive success (Obeso, 2002) and represents one of the three key components of plant performance - growth, reproduction, and survival (Violle et al., 2007; Dias et al., 2020).

### 2.3 Biotic and abiotic factors along the latitudinal distribution of species

**Fig. 2:**
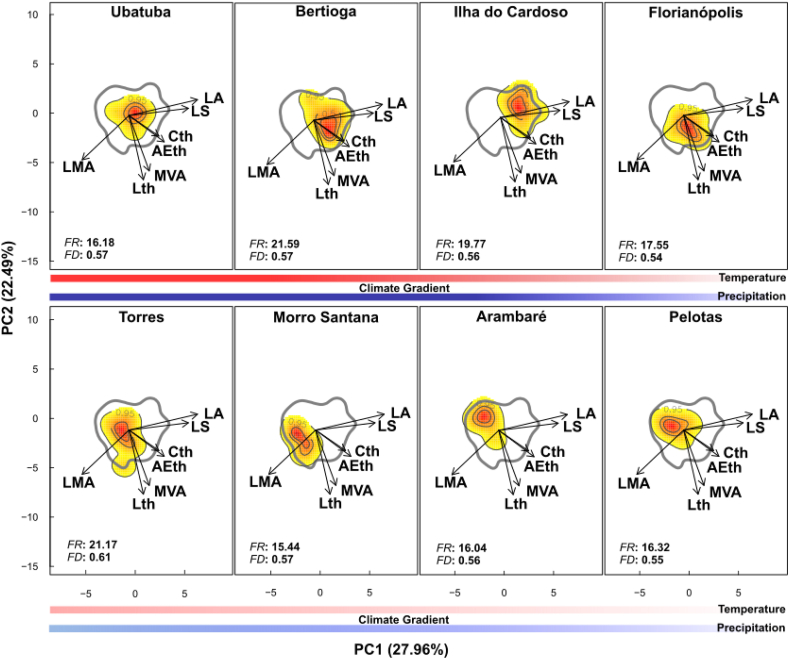
The Functional Space (FS) was constructed using a Principal Component Analysis (PCA) on leaf functional traits measured from all *Epidendrum fulgens* individuals across populations along a climatic gradient (mean annual temperature – MAT, and mean annual precipitation – MAP), ranging from the warm and humid periphery (starting in Ubatuba, SP) to the cold and dry periphery (Pelotas, RS). The thick gray outline in each panel represents the species-level FS, based on the entire trait dataset. Population-specific FSs are nested within this global space and are shown using yellow-to-red color gradients, where red indicates areas with higher probability density of individual trait combinations. This visualization highlights how each population occupies and varies within the shared functional space. Abbreviations: FR - Functional richness; FD - Functional divergence; AEth - adaxial epidermis thickness; Cth - cuticle thickness; LA-leaf area; LMA - leaf dry mass per unit area; LS - leaf succulence; Lth - leaf thickness; MVA - metaxylem vessel area.

For each locality, we obtained bioclimatic variables with a 30 s resolution from the WorldClim database (Fick and Hijmans, 2017). We then selected Bio1 (Mean Annual Temperature) and Annual Precipitation (Bio12) for further analysis, as it captured the major climatic gradient across our study sites (Fig. 2; de Mattos et al., 2023).

To estimate the impact of biotic (species interactions as competition) and abiotic (environmental filtering) factors on population performances, we assessed the phylogenetic structures (Webb et al., 2002) of plant communities surrounding each *Epidendrum fulgens* population. We calculated the evolutionary relationships among co-occurring species in each plant community by means of the mean pairwise distance (MPD, Webb et al., 2002; Kembel et al., 2010). We first constructed a presence-absence matrix of the plant community associated with each *E*. *fulgens* population (Fig. 1), using species occurrence records from the SpeciesLink database (http://splink.cria.org.br/). For each population, we retrieved records within the corresponding municipality and applied a spatial filter to retain only species with documented occurrences within a 5-km radius of the geographic centroid of the population’s locality. This spatial window was used to approximate the local plant assemblage potentially co-occurring with *E*. *fulgens*. Finally, to ensure that only taxa occupying the same restrictive habitats as *E*. *fulgens* were retained, we applied an environmental filter with the R package “flora” (v.0.3.4), preserving exclusively those species documented in *Restingas* or rocky outcrop habitats.

After the construction of the data set of plant communities, we performed phylogenetic analysis. We first constructed a phylogenetic tree (supertree) using the “*U.PhyloMaker*” package (Jin and Qian, 2023), and we built a cophenetic distance matrix over millions of years using the “*ape*” package. We then analyzed the phylogenetic structure keeping community richness as a fixed parameter using the “*picante*” package (v.1.8.2). Finally, we focused on calculating the standardized effect size of MPD (hereafter only MPD) within plant communities across each studied locality. Here, we use the null model by randomly assigning the occurrence matrix to samples while maintaining the richness of the sample species (Webb et al., 2008). Generally, significant positive MPD values (phylogenetic overdispersion) point towards competitive interactions as the primary ecological process that drove species co-occurrence within the community. In general, competitive exclusion is expected to reduce the occurrence of closely related taxa within communities, resulting in positive MPD values. In contrast, negative MPD values indicate phylogenetic clustering driven by habitat filtering, where conserved traits influence species’ tolerance to abiotic conditions (Webb et al., 2008).

### 2.4 Data analysis

#### 2.4.1 Assessing functional space across populations

To quantify within-population variation in functional strategies, we used a two-step workflow implemented in the “*funspace*” R package (Carmona et al., 2024). First, we established a global, species-level functional trait space by pooling imputed leaf-trait data from all *Epidendrum fulgens* individuals. We fitted a multivariate probability distribution to this combined dataset, defined its 95% quantile hypervolume, and then performed a principal component analysis (PCA) on the imputed traits to identify the primary axes of inter-individual variation (retaining PC1 and PC2). The resulting global PCA yields a common biplot embedding all individuals (and, by implication, all populations). We then quantified each population’s functional-strategy variance by projecting its constituent individuals into this shared PC1–PC2 space and estimating the multivariate probability distribution of their trait values within the pre-defined 95% hypervolume. From these distributions, we derived both functional richness (FR), the proportion of the species-level functional space occupied by each population, and functional divergence (FD), which reflects the extent to which individuals within a population differ in their trait combinations (Carmona et al., 2024).

This approach allows visualizing and comparing population-level trait distributions within a standardized framework. The position and spread of each population’s probability distribution in the PCA space reflect the dominant trait combinations and their variability relative to the global axes. As such, shifts in these distributions can be interpreted as directional trends toward more acquisitive or conservative strategies, depending on their alignment with specific trait loadings.

#### 2.4.2 Effects of IPD, ITV plus biotic and abiotic factors on the performance of *Epidendrum fulgens*

To evaluate the role of intraspecific phenotypic dissimilarity (IPD) on the performance of species populations across their latitudinal distribution, we initially calculated IPD at the population level by comparing the differences in all trait values between all pairs of *Epidendrum fulgens* populations. We computed IPD as the Phenotypic Dissimilarity Index (PhD) proposed by Puglielli et al. (2022). PhD calculates the average phenotypic dissimilarity between individuals from different populations, accounting for the variability of characteristics within each population. Here, we use the PhD to calculate the intraspecific phenotypic dissimilarity of individuals between different populations, using a Euclidean distance matrix for all functional traits (LMA, LA, Lth, Cth, MVA, AEth and LS), with standardized trait values. Initially, nonparametric tests (Kruskal–Wallis test) and paired Wilcoxon tests were conducted to discern differences in both PhD and performance across populations.

We then assessed the effects of IPD, environmental factors and species competition, along with their interactions, on performance of *Epidendrum fulgens*, by using generalized linear models (GLMs). Here, we used IPD as the mean value of the PhD index for all traits for each population. We first selected the model that best explained these variables in the species’ performance, using fewer variables and the lowest Akaike Information Criterion (AIC) value (Bozdogan, 1987). This resulted in a model (AIC = 1642 compared to AIC = 1741 for the full model) that included IPD, MPD, mean annual temperature, and interactions between IPD and temperature and between IPD and MPD (Table S2). We used a Poisson distribution, with dispersion fixed at one and the number of parameters equal to the number of coefficients, for GLM construction.

Finally, to address how the intrapopulation variability of each trait influences population performance, we first quantified the intrapopulation trait variability (ITV) within populations using a novel approach for calculating the phenotypic dissimilarity index. We adapt the PhD index to assess pairwise dissimilarity, employing a Euclidean distance matrix for each functional trait: LMA, LA, Lth, Cth, MVA, AEth, and LS, with values standardized for all individuals within each distinct population. Using the ITV of each trait as a predictor variable, we developed a generalized linear model (GLM) based on the number of fruits. We selected the model with the best explanatory power while containing the fewest variables (as described above), resulting in a model with all traits and without interactions. We used GLM construction as described above. We also conducted nonparametric tests (Kruskal–Wallis test) and paired Wilcoxon tests to identify differences in ITV values of each functional trait among populations.

## 3 Results

### 3.1 Effect of latitudinal variation on populations' functional strategies

The functional space of *Epidendrum fulgens* (Fig. 2) was characterized by two principal dimensions derived from a global Principal Component Analysis (PCA) including all individuals from all populations, accounting for 50.45% of the total variance in functional traits. PC1 explained 27.96%, while PC2 accounted for 22.49%. PC1 was primarily driven by variations in leaf area (LA), leaf succulence (LS), leaf dry mass per unit area (LMA), adaxial epidermis thickness (AEth), and cuticle thickness (Cth), whereas PC2 reflected variation in leaf thickness (Lth) and metaxylem vessel area (MVA).

Population-level functional strategies were inferred by examining the position of each population's multivariate trait distribution within the species’ PCA space (Fig. 2). A pattern of variation in functional strategies was observed across populations along the latitudinal gradient of *Epidendrum fulgens*. Populations in warmer and wetter regions tended to exhibit acquisitive strategies, characterized by higher values of LA, LS, Cth, and AEth. In contrast, populations in colder and drier climates showed more conservative strategies, with lower values for these traits, higher LMA and MVA, and reduced Lth. However, functional richness (FR) did not show a consistent pattern along the latitudinal gradient. In the warmer region, the Ubatuba population exhibited low FR, whereas Bertioga showed the highest values. In the mid-latitude region, Torres presented high FR, while Morro Santana had the lowest. In the colder southernmost region, Arambaré and Pelotas displayed intermediate FR.

### 3.2 Influence of abiotic and biotic factors on *Epidendrum fulgens* performance

#### 3.2.1 Contrasting performance and IPD across *Epidendrum fulgens* populations

**Fig. 3:**
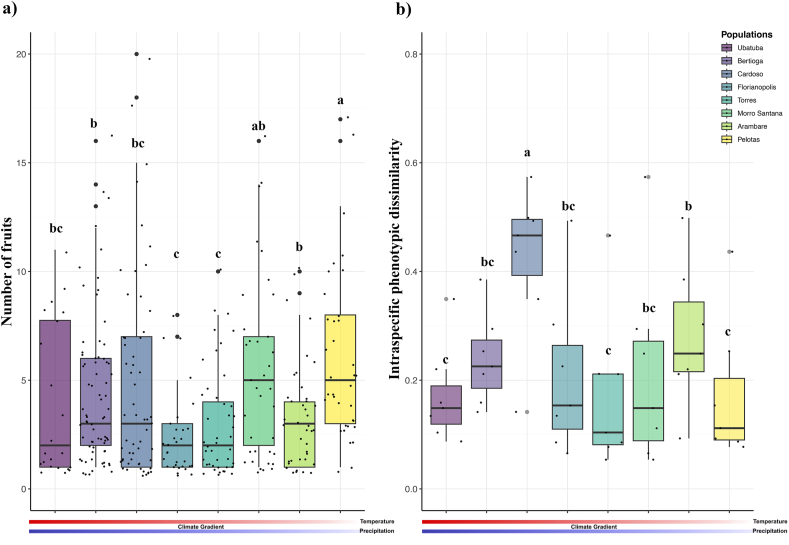
Variation in fruit set per plant (a) and in the intraspecific phenotypic dissimilarity (IPD) (b) across populations of *Epidendrum fulgens*, distributed along a climatic gradient on the Brazilian coast. The letters above the box plots denote statistically significant differences between populations, as determined by post-hoc multiple comparison tests.

We observed statistically significant variations in fruit set and intraspecific phenotypic dissimilarity (IPD) across populations (Fig. 3a and b). However, these variation patterns were neither parallel nor related to the latitudinal gradient. The lowest levels of IPD were found in populations at the peripheries of the latitudinal gradient. Specifically, Pelotas population, in the coldest region, exhibited the highest fruit set, while the Cardoso population, in the warmer region, showed the highest level of IPD. In contrast, populations located in the middle of the latitudinal gradient, such as Florianópolis and Torres, showed the lowest fruit set.

#### 3.2.2 Ecological factors that shape species performance

**Fig. 4:**
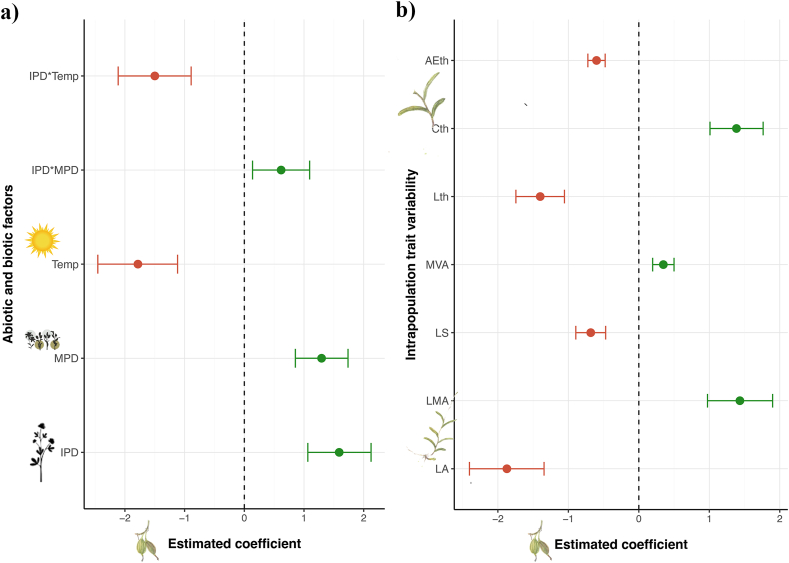
The effects of abiotic and biotic factors (a), along with their interactions, and intrapopulation variability of functional traits (b) were examined in relation to the estimated number of fruits of *Epidendrum fulgens* in its latitudinal distribution along the Brazilian coast. Models were constructed using generalized linear models. Abbreviations: IPD - Intraspecific phenotypic dissimilarity computed as phenotypic dissimilarity for all traits in each population; MPD - Mean pairwise distance; Temp - mean annual temperature (Bio1); AEth - adaxial epidermis thickness; Cth - cuticle thickness; LA - leaf area; and LMA - leaf mass per area; LS – leaf succulence. The factors highlighted in red indicate a negative relationship with performance, the traits highlighted in green indicate a positive relationship with performance, and those in gray indicate no discernible relationships.

Performance of *Epidendrum fulgens* was positively related to intraspecific phenotypic dissimilarity (IPD), mean pairwise distance (MPD), and the interaction between IPD and MPD, as well as mean annual temperature (Bio1). Our model (Fig. 4a) explains a substantial portion of the variation in performance, with an R^2^ value of 0.348. IPD and MPD had the most significant positive effects on the performance of *E*. *fulgens* individuals. In terms of climatic factors, higher temperatures were negatively correlated with individual performance (*p* < 0.001), suggesting that elevated temperatures negatively impact the species’ performance. Additionally, interactions between MPD, temperature, and IPD reduced the significance of IPD on performance.

The performance of *Epidendrum fulgens* populations was both positively and negatively influenced by ITV within populations across individual functional traits (Fig. 4b). The ITV model explained 38% of the variation in species performance. Among anatomical traits, Cth and MV showed positive correlations with the fruit set, whereas AEth and Lth exhibited negative correlations. Regarding integrative traits, LMA displayed a strong positive correlation with performance, while LS and LA were negatively correlated, with LA having the most pronounced negative effect on the fruit set. Overall, we observed significant differences (*P* < 0.05) in ITV across populations of *E*. *fulgens* along the latitudinal gradient (Fig. S2), with certain populations exhibiting high ITV values (greater than 0.3) for LA, LMA, and LS.

## 4 Discussion

Our study offers a novel population-level perspective on how species adapt to varying environmental conditions and how these adaptations influence their distribution and performance (Willi and Van Buskirk, 2019). We found that populations of the widely distributed *Epidendrum fulgens* occupy distinct regions of the species’ overall functional space, suggesting potential effects of local adaptation (Pelletier et al., 2023). Higher intrapopulation phenotypic dissimilarity (IPD) was associated with better population performance, while interspecific competition and abiotic factors influenced species performance in various ways. Interestingly, our results indicate that conditions typically considered stressful, such as elevated interspecific competition and lower temperatures (Valladares et al., 2007; Aschehoug et al., 2016), can actually enhance species performance, as well as how *E*. *fulgens* populations are well adapted to the conditions they inhabit. We also discovered that higher IPD reduces the strong negative relationship between temperature and population performance. Additionally, we found that populations’ performance is significantly influenced by ITV within populations, with specific traits enhancing performance, while others are associated with reduced performance. Collectively, our findings underscore the critical role of intrapopulation trait variability in shaping functional strategies and overall performance of species across environmental gradients and their importance in uncovering the mechanisms behind plant distribution and adaptation.

### 4.1 Changes in the functional strategy of populations along the latitudinal gradient

We found that *Epidendrum fulgens* functional richness did not vary markedly among populations; however, populations occupied distinct regions within the species’ overall functional space, suggesting the expression of different phenotypes along the latitudinal gradient. Notably, individuals from each population formed clusters around specific traits (Fig. 2), indicating the presence of unique phenotypic profiles, potentially shaped by local adaptation (Münzbergová et al., 2017). Such differentiation is expected given the species’ wide geographic distribution, which spans significant climatic variation and diverse biotic interactions (de Mattos et al., 2023). Plant functional traits are known to be effective in explaining species distributions in relation to environmental conditions (Violle et al., 2012; Díaz et al., 2016), and variation within populations contributes to their ecological success (Bolnick et al., 2011; Jung et al., 2010). Thus, our results indicate that, although functional richness remains relatively consistent across populations, *E*. *fulgens* exhibits pronounced divergence in trait composition, likely as a consequence of local adaptation to heterogeneous environmental conditions.

As expected, changes in functional trait trade-offs can drive variation in resource acquisition and allocation strategies among populations. We found that harsh environments characterized by lower temperatures and reduced precipitation prompted populations to adopt more conservative strategies (Violle et al., 2007; Díaz et al., 2016). Specifically, populations in colder and drier habitats exhibited a higher leaf mass per area (LMA) and metaxylem vessel area (MVA), traits associated with increased water use efficiency and survival under stress (Poorter et al., 2009; Li et al., 2023). On the contrary, populations in warmer, wetter regions showed traits such as greater leaf area (LA), leaf succulence (LS), and adaxial cuticle thickness (Cth), which correlate with rapid growth and resilience to variable water availability (Wright et al., 2004; Pérez-Harguindeguy et al., 2016). An unexpected finding was the larger MVA in drier areas at the southern periphery of the *Epidendrum fulgens* distribution, contrary to previous studies suggesting that vessel size is typically reduced to mitigate embolism risk; however, most of which focused on woody species (see, e.g., Levionnois et al., 2021; Dória et al., 2022; Isasa et al., 2023). We propose that the larger MVA in *E*. *fulgens* reflects a strategy that prioritizes water efficiency, as this species possesses highly succulent leaves, a relatively thick cuticle, and velamen-covered roots typical of orchids, indicating a trade-off between hydraulic safety and efficiency (Li et al., 2023).

### 4.2 How intrapopulation trait variability, species interactions, and climate affect population performance?

IPD and performance varied significantly among *Epidendrum fulgens* populations, although without a consistent latitudinal pattern. Still, populations with higher functional dissimilarity tended to perform better, suggesting environment-specific optimal trait combinations (Laughlin and Messier, 2015; Dias et al., 2020), and the contrasts in IPD among peripheral populations in colder outcrops supported both stress-induced and stress-reduced variability (Helsen et al., 2017). In general, higher IPD was positively associated with performance, highlighting its role in enabling broad environmental tolerance. Phenotypic plasticity supports individual survival in heterogeneous environments (Edelaar et al., 2017; Tudor et al., 2024), and our results indicate that variation in population phenotype can buffer negative climatic effects along the gradient (Dias et al., 2020; Kamimura et al., 2023). Also, niche models show that *E*. *fulgens* faces climate limitations in colder regions but thrives in warmer, pollinator-rich areas (de Mattos et al., 2023). Our results highlight the importance of population-level trait divergence in increasing phenotypic variability across environmental gradients (Camps et al., 2021; Pfeilsticker et al., 2021), reinforcing the value of *E*. *fulgens* as a model species for investigating population differentiation and ecological adaptation (Pinheiro and Cozzolino, 2013; Sujii et al., 2019; Leal et al., 2020; de Lima et al., 2023; de Mattos et al., 2023; de Lima et al., 2024b, de Lima and da Silva, 2024a).

Interestingly, MPD values were positively correlated with performance, suggesting that higher competition levels may enhance the reproductive success of *Epidendrum fulgens*. In plant communities, both biotic and abiotic factors strongly influence species’ distribution ranges (Willi and Van Buskirk, 2019), with interactions among individuals ranging from competition for limited resources to facilitation, often shaped by local environmental conditions (Callaway, 2007; Aschehoug et al., 2016). These interactions may impact different aspects of plant fitness in contrasting ways (Goldberg and Landa 1991). For instance, while competition can reduce components like seed production, co-occurrence with other species may increase pollinator visitation in different habitats, improving reproductive success (Hurtado et al., 2023). This ‘magnet species effect’ may increase rewarding species by pollination of neighboring plants (Thomson 1978). Nonrewarding orchids, including *E*. *fulgens*, may benefit similarly when co-occurring with nectar-producing species (Alexandersson and Ågren 1996; Peter and Johnson 2008). Additionally, butterfly pollinators of *E*. *fulgens* are more abundant in northern populations (de Mattos et al., 2023), where MPD values are higher (Fig. S2). This suggests that increased plant–plant interaction may boost pollinator attraction and fruit set, enhancing colonization potential and population resilience in competitive or fragmented habitats (García 2008).

Contrary to our hypothesis that higher ITV within populations would correlate with better performance, we found that variability in certain functional traits within populations can have both positive (e.g., Cth, MVA, LMA) and negative effects (e.g., AEth, Lth, LS, LA) on performance. While phenotypic variability can expand ecological breadth in diverse environments, particularly in habitats with frequent disturbances (Schlichting and Levin, 1986), it does not always benefit individuals (Bolnick et al., 2003, 2011). Some traits, like Cth and Lth, may have optimal values in specific environments, such as arid areas with drought and nutrient limitations (Poorter et al., 2009), and high variability in these traits can hinder performance in such conditions. Additionally, large leaves tend to thrive in warm, sunny, and humid areas, while smaller leaves are more common in arid, warm, and sunny environments (Wright et al., 2017). Nonetheless, high trait variability can increase population resilience to climate change-related stresses, demonstrating that trait variability plays a complex role in species performance, adaptation, and distribution.

## 5 Conclusions

In a nutshell, our study provides evidence that (i) incongruences in IPD values among populations in the species' distribution periphery indicate both stress-reduced and stress-induced variability; (ii) populations can occupy distinct regions of the species’ functional space, reflecting adaptations to their specific environments; (iii) higher IPD is generally associated with better population performance and decrease the negative effects of temperature changes, suggesting its role in local adaptation; (iv) ITV within populations is not consistently linked to better performance, as variability in certain traits has both positive and negative impacts; and (v) the interplay between intrapopulation trait variability, environmental conditions, and species interactions underscores the complex nature of plant adaptation and distribution. Future research should focus on exploring the mechanistic links between ITV within populations and fitness in different environmental contexts, particularly in climate change scenarios, to improve our understanding of plant resilience and adaptive strategies (Westerband et al., 2021). Additionally, examining the role of trait variability in facilitating species interactions and community dynamics will provide deeper insights into the ecological and evolutionary processes shaping plant populations and communities, potentially affecting even the interaction network (Berg and Ellers, 2010; Yang et al., 2024).

## CRediT authorship contribution statement

**Welington L. Sachetti:** Writing – review & editing, Writing – original draft, Methodology, Funding acquisition, Data curation. **Vitor de A. Kamimura:** Writing – review & editing, Writing – original draft, Visualization, Project administration, Methodology, Investigation, Formal analysis, Data curation, Conceptualization. **Juliana L.S. Mayer:** Writing – review & editing, Writing – original draft. **Beatriz L. Arida:** Writing – review & editing, Writing – original draft, Data curation. **Thales M. de Lima:** Writing – review & editing, Writing – original draft, Data curation. **Diego S. Graciano:** Writing – review & editing, Writing – original draft, Data curation. **Fábio Pinheiro:** Writing – review & editing, Writing – original draft, Project administration, Methodology, Funding acquisition, Formal analysis, Data curation, Conceptualization.

## Declaration of competing interest

The authors declare no conflict of interest.

## Figures and Tables

**figs1:**
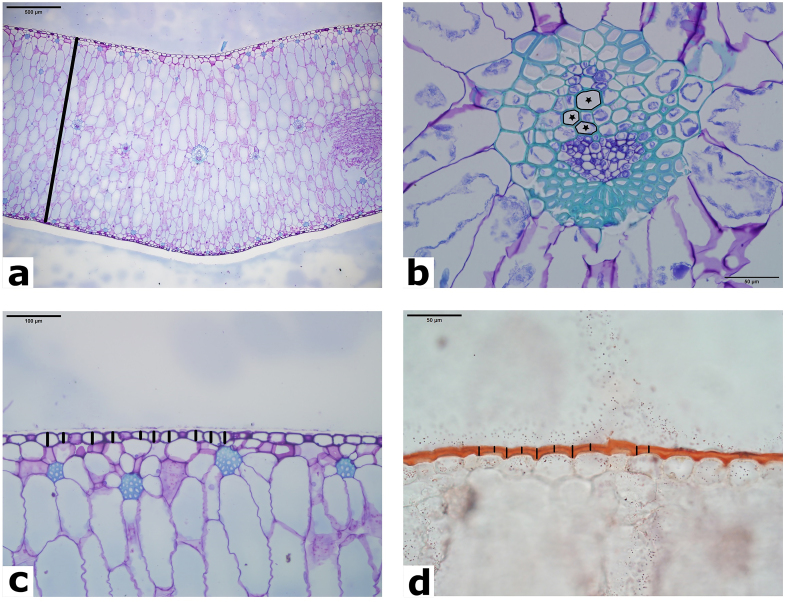


**figs2:**