# Supplementary material for: We are not all the same: The role of intrapopulation trait variability in shaping functional strategy and performance of widespread species
Source: Plant Divers. 2025 Jun 30;48(3):576–85. doi: 10.1016/j.pld.2025.06.008 (PMC13250299; doi:10.1016/j.pld.2025.06.008)
Supplement: Multimedia component 5 [file mmc5.docx]

**Appendices**

**Table S1** - Environmental conditions of eight populations of *Epidendrum fulgens* along its latitudinal distribution on the Brazilian coast. Köppen climate classification, mean annual precipitation (MAP), mean annual temperature (MAT), and soil properties.

| **Locality (Population)** | **Latitude** | **Longitude** | **Climatic and soil characteristics** |
| --- | --- | --- | --- |
| **Ubatuba** | 23°22'45.23" S | 44°57'28.34" W | Af (Köppen), MAP: 2,650 mm, MAT: 21.9 °C.  Sandy soil, neutral to slightly acidic. |
| **Bertioga** | 23°46'37.92" S | 45°57'24.83" W | Af (Köppen), MAP: 3,207 mm, MAT: 24 °C.  Sandy and acidic soil. |
| **Ilha do Cardoso** | 25°4'9.89" S | 47°54'38.75" W | Af (Köppen), MAP: 2,248 mm, MAT: 21.3 °C.  Sandy, acidic soil with marine influence. |
| **Florianópolis** | 27°37'55.22" S | 48°27'28.04" W | Cfa (Köppen), MAP: 1,506 mm, MAT: 20.1 °C.  Sandy, acidic soil with marine influence. |
| **Torres** | 29°22'41.29" S | 49°45'17.25" W | Cfa (Köppen), MAP: 1,789 mm, MAT: 19.7 °C.  Sandy, acidic soil. |
| **Morro Santana** | 30°3'23.05" S | 51°7'36.12" W | Cfa (Köppen), MAP: 1,580 mm, MAT: 19.7 °C.  Shallow, rocky, acidic soil. |
| **Arambaré** | 30°54'4.51" S | 51°29'20.03" W | Cfa (Köppen), MAP: 1,580 mm, MAT: 19.7 °C.  Shallow, rocky, acidic soil. |
| **Pelotas** | 31°28'30.98" S | 52°25'32.01" W | Cfa (Köppen), MAP: 1,425 mm, MAT: 18.8 °C.  Acidic soil on granite outcrops. |

**Table S2** - The climatic variables analyzed include Mean Annual Temperature (Bio 1), Temperature Seasonality (Bio 4), Mean Temperature of the Coldest Quarter (Bio 11), Mean Annual Precipitation (Bio 12), Precipitation of the Wettest Month (Bio 13), Precipitation of the Driest Quarter (Bio 17), Precipitation of the Warmest Quarter (Bio 18), and Precipitation of the Coldest Quarter (Bio 19) across eight populations of *Epidendrum fulgens* along its latitudinal distribution on the Brazilian coast. Additionally, Mean Phylogenetic Distance (MPD) Z and P-values, as well as Mean Phenotypic Dissimilarity (PhD), were assessed.

| Locality (Population) | Bio1 | Bio4 | Bio11 | Bio12 | Bio13 | Bio17 | Bio18 | MPD (Z-value) | MPD (P-value) | Mean PhD | Mean number of fruits |
| --- | --- | --- | --- | --- | --- | --- | --- | --- | --- | --- | --- |
| Ubatuba | 22.39 | 240.06 | 19.51 | 2,537 | 321 | 317 | 940 | 1.44 | 0.93 | 0.13 | 2.27 |
| Bertioga | 22.26 | 244.31 | 19.26 | 2,935 | 356 | 391 | 1,021 | 1.08 | 0.87 | 0.19 | 3.53 |
| Ilha do Cardoso | 22.36 | 287.63 | 18.89 | 2,162 | 319 | 277 | 893 | 0.66 | 0.74 | 0.36 | 3.41 |
| Florianópolis | 20 | 312.25 | 16.27 | 1,575 | 177 | 324 | 511 | -0.76 | 0.74 | 0.17 | 1.35 |
| Torres | 19.26 | 332.86 | 15.21 | 1,415 | 146 | 315 | 407 | 0.4 | 0.65 | 0.14 | 2.45 |
| Morro Santana | 19.58 | 358.78 | 15.16 | 1,519 | 146 | 349 | 410 | 0.97 | 0.84 | 0.18 | 4.29 |
| Arambaré | 19.38 | 379.63 | 14.69 | 1,384 | 132 | 300 | 363 | -2.27 | 0.01 | 0.2 | 2.43 |
| Pelotas | 18.41 | 397.71 | 13.5 | 1,434 | 143 | 296 | 343 | 1.13 | 0.88 | 0.14 | 6.03 |


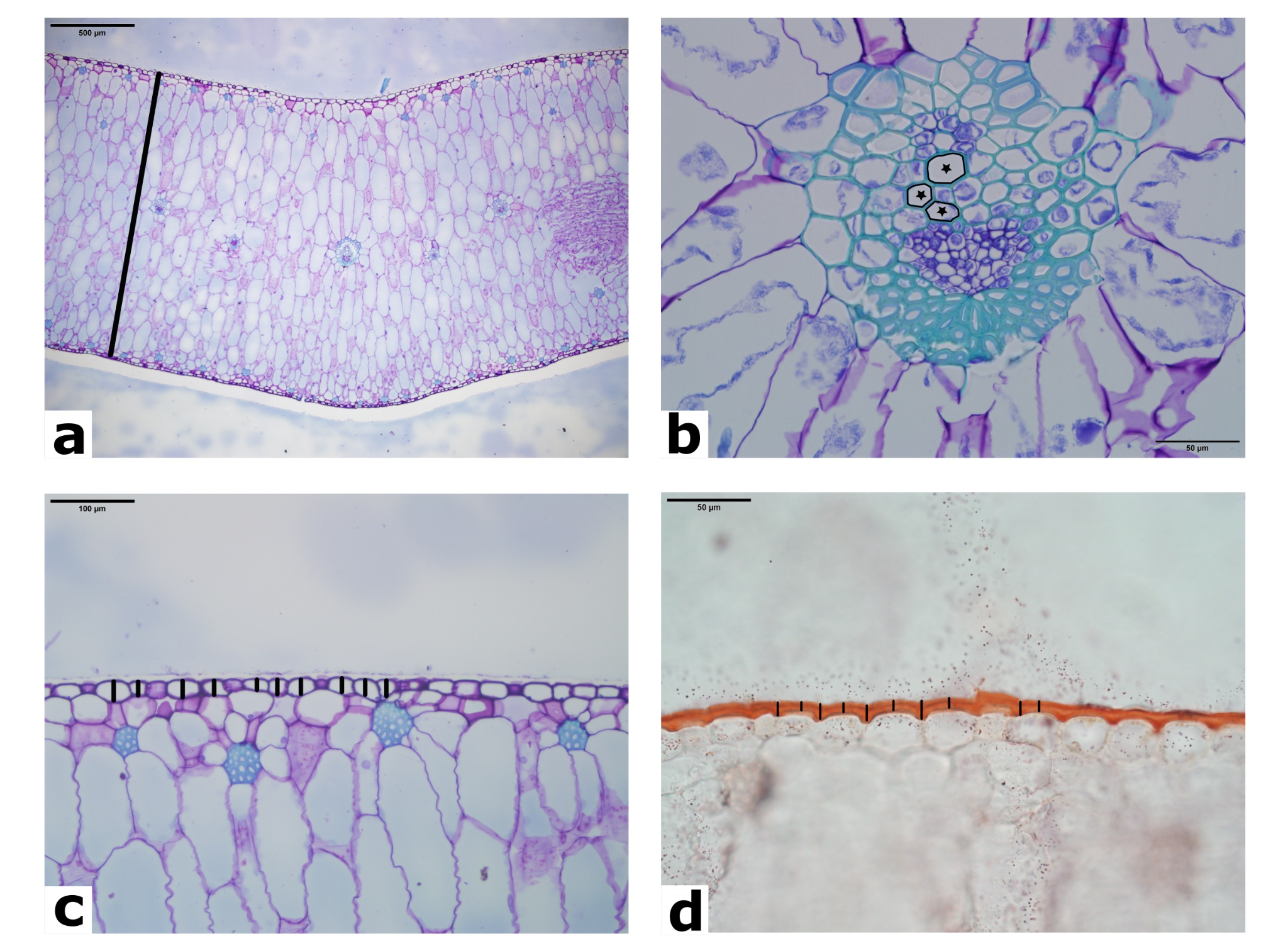


**Figure S1.** Anatomical traits measured for eight populations of *Epidendrum fulgens* along its latitudinal distribution on the Brazilian coast. The traits measured were (a) leaf thickness, with black bars indicating the measured regions; (b) metaxylem vessel area, with asterisks marking the three largest metaxylem vessels; (c) adaxial leaf thickness assessed from ten cells to calculate the average thickness; and (d) adaxial cuticle thickness along the leaf surface, measured from ten different regions to determine the average cuticle thickness.


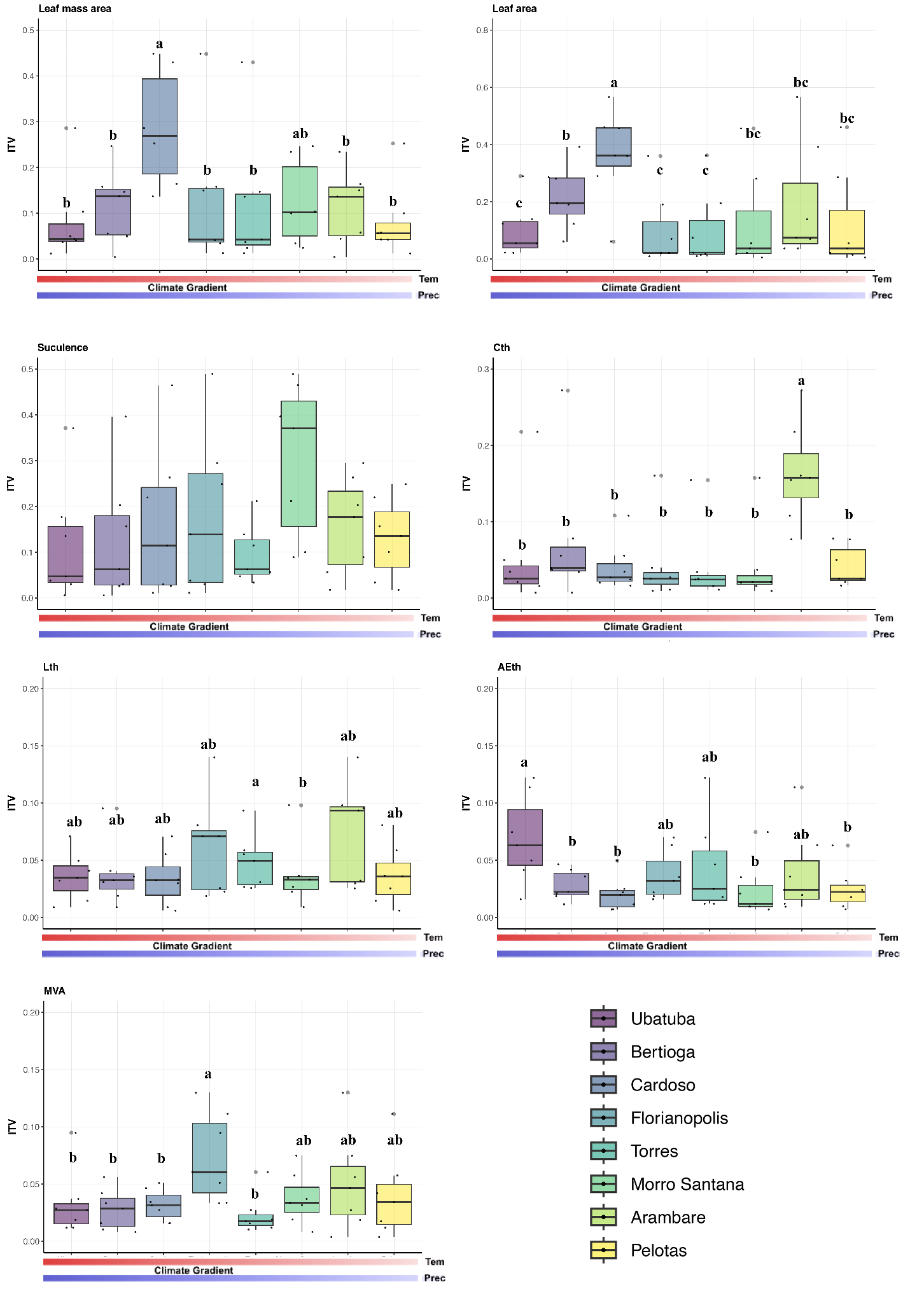


**Figure S2**. Intraspecific trait variation within populations (ITV) was analyzed for seven leaf traits across eight populations of *Epidendrum fulgens* along its latitudinal distribution on the Brazilian coast. The red band represents the temperature gradient, while the blue band indicates the precipitation gradient, both decreasing towards the species' southern distribution. Different letters denote significant differences (P < 0.01) between populations. The leaf traits examined include leaf mass per area (LMA), leaf area (LA), succulence (SL), Adaxial Cuticle Thickness (Cth), Leaf thickness (Lth), Adaxial Epidermis Thickness (AEth), and Metaxylem Vessel Area (MVA) according to the methods described in the Table S. 1.
